# Counteracting breast-cancer induced immune suppression by reactivating lymph node-resident conventional dendritic cells (LNR-cDC)

**DOI:** 10.1186/2051-1426-3-S2-P276

**Published:** 2015-11-04

**Authors:** Rieneke van de Ven, Kim van Pul, Shaghayegh Aliabadi, Petrousjka van den Tol, Lisette A te Velde, Emiel JTh Rutgers, Ronald JCLM Vuylsteke, Hein BAC Stockmann, Christopher Dubay, Chris Harrington, Bernard A Fox, Tanja D de Gruijl

**Affiliations:** 1 VU University medical center, Cancer Center Amsterdam, Amsterdam, Netherlands; 2 Robert W. Franz Cancer Research center at the Earle A. Chiles Research Institute (EACRI), Providence Cancer Center, Portland, OR, USA; 3 Antoni van Leeuwenhoek Hospital, Netherlands Cancer Institute, Amsterdam, Netherlands; 4 Spaarne Gasthuis, Haarlem, Netherlands; 5 Oregon Health and Sciences University (OHSU), Portland, OR, USA; 6 Earle A. Chiles Research Institute, Portland, OR, USA

## 

Breast cancer (BrC)-derived factors inhibit proper development and activation of dendritic cells (DC), which in turn promote the expansion of regulatory T cells (Tregs), thus contributing to tumor progression and spread. In two separate BrC sentinel lymph node (SLN) cohorts, one from the EACRI in Portland and one from the VUmc in Amsterdam, we found metastasis-related immune suppression of CD11c+CD14-lymph node-resident conventional DC (LNR-cDC).

Genome-wide microarray analyses on FACS sorted migratory CD1a+ cDC, LNR-cDC and CD14+ LNR-cDC isolated from breast-draining LN were performed. A focused analysis of c-type lectin receptors (CLR) and transcription factor genes associated with Ag uptake and cross-presentation, showed LNR-cDC to express relatively high transcript levels of Interferon-regulatory factor 8 (IRF8), chemokine receptor 1 (XCR1), BDCA3/thrombomodulin, and, remarkably, of Langerin, CLEC7A and CLEC4A. These are interesting observations, since these are all factors previously linked to efficient antigen cross-presentation and cross-priming of CD8^+^ T cells.

The EACRI cohort (n=38) included LN from ductal carcinoma in situ (DCIS), tumor-negative SLN (SLN-) and metastasis-positive SLN (SLN+), analyzed with a 12-parameter flow cytometry panel. The VUmc cohort included healthy, prophylactically removed breast LN (HLN, n=16), SLN- and SLN+ (n=40). In both cohorts, there was significantly reduced activation of LNR-cDC, but not of migratory cDC, in SLN-versus DCIS/HLN with further reductions in SLN+. Notably, reduction in activation of LNR-cDC went hand-in-hand with increased Treg frequencies.

We next explored the possibility to counteract this BrC-related suppression in SLN *ex vivo*. The combined activity of CpG-B and the JAK2/STAT3 inhibitor (STAT3i) AG490 resulted in increased activation of LNR-plasmacytoid DC and LNR-cDC. It also promoted production of IFNγ by CD8^+^ T cells, reduced production of IL-4 by CD4^+^ T cells, and decreased rates of suppressive Tregs, which were slightly elevated by CpG-B alone. Finally, the combination of CpG-B and STAT3i increased tumor-specific effector T cell responses as measured by IFNγ ELISPOT assay after *in vitro* restimulation against a pool of overlapping 15-mer peptides, covering the sequence of the BrC–associated antigen Mammaglobin-A.

Combined, our data show that BrC-mediated suppression in SLN is primarily directed against the LNR-cDC subset (with particular cross-presenting characteristics and abilities), rather than CD1a^+^ cDC subsets that migrate to the SLN from local tissues. Stimulation with CpG-B and STAT3i could reactivate these suppressed LNR-cDC ex vivo, thereby restoring type-1 mediated anti-tumor immunity. In view of increasing evidence that immune-regulated pathways influence response to (neo)adjuvant chemotherapy, we anticipate clinical benefit of combination therapy with this immune-stimulatory cocktail.

